# Exploring the Impact of Personality Trait Clusters on the Quality of Life of Breast Cancer Survivors: An 18‐Month Prospective Follow‐Up Study

**DOI:** 10.1002/cam4.70842

**Published:** 2025-05-02

**Authors:** In Mok Song, Eun Young Cho, Ji Hyun Baek, Se Kyung Lee

**Affiliations:** ^1^ Department of Psychiatry, Samsung Medical Center Sungkyunkwan University School of Medicine Seoul Korea; ^2^ Department of Psychiatry Maritime Medical Center Changwon Korea; ^3^ Samsung Biomedical Research Institute Seoul Korea; ^4^ Division of Breast Surgery, Department of Surgery, Samsung Medical Center Sungkyunkwan University School of Medicine Seoul Korea

**Keywords:** breast cancer survivor, neuroticism, personality, polygenic risk score, psycho‐oncology, quality of life

## Abstract

**Objective:**

To investigate the impact of personality trait clusters on the quality of life (QoL) of breast cancer survivors (BCS) during the first 18 months following diagnosis.

**Methods:**

A cohort of 476 newly diagnosed breast cancer patients was recruited between January 2017 and August 2018 from a single academic hospital in Seoul, Korea. Five‐factor models of personality traits were assessed at baseline. QoL evaluations were performed prior to surgery and up to 18 months post‐surgery. K‐means clustering analysis was employed to construct personality clusters. Long‐term QoL trajectories in BCS were compared between clusters, adjusting for individual resilience. Furthermore, a polygenic risk score (PRS) for neuroticism was calculated, exploring its relationships with neuroticism and personality trait clusters identified in this study.

**Results:**

Cluster analysis suggested that a two‐cluster model was more appropriate than a three‐cluster model. The two clusters were characterized by (1) low neuroticism and high scores in the other four traits, and (2) high neuroticism and low scores in the other four traits. Patients in cluster 2 exhibited significantly lower baseline QoL scores compared to those in other clusters, from baseline through 18 months post‐surgery. The PRS for neuroticism showed a significant association with neuroticism scores (*p* = 0.032) after adjusting for age and depression scores. No significant differences in PRS were observed between the clusters. Additionally, the PRS for neuroticism was not significantly associated with QoL.

**Conclusion:**

Our findings underscore the influence of individual personality traits on long‐term QoL in BCS. These results suggest the potential for targeted interventions to enhance long‐term QoL based on personalized personality profiles.

## 1 Background

Breast cancer has become the most prevalent cancer among women globally, with an astounding 2.26 million cases reported in 2020 [1]. Advances in medicine have significantly improved survival rates and life expectancy, leading to a substantial increase in the number of breast cancer survivors (BCS) [2]. Consequently, the quality of life (QoL) and mental health of breast cancer survivors have become increasingly vital issues [3].

QoL after cancer diagnosis varies. To improve the QoL of BCS, individualized approaches based on factors influencing QoL are essential. In addition to the diverse factors linked with breast cancer itself and its treatment [4, 5, 6, 7], an individual's personality traits can impact their QoL. Personality traits are relatively enduring patterns of thoughts, feelings, and behaviors that manifest as consistent responses under specific circumstances [8]. These traits influence how individuals adapt to life events, including the unique long‐term physical and psychological challenges faced by breast cancer survivors. Recognizing these psychological factors is crucial for identifying at‐risk individuals and devising personalized interventions.

Personality traits are multidimensional. Higher scores on agreeableness, openness to experience, extraversion, and conscientiousness are linked to a better perception of health, thus improving overall QoL. Conversely, high levels of neuroticism are negatively associated with psychological outcomes [9, 10, 11, 12]. Many researchers advocate for a holistic, person‐centered approach to personality traits rather than focusing solely on the impact of individual traits [13, 14].

Resilience is the capacity to preserve mental health during adverse situations. It is considered a trait of an individual and is influenced by their personality. Moreover, resilience affects QoL in addition to personality traits. It impacts various aspects of QoL. Breast cancer survivors who display greater resilience may significantly benefit from enhanced QoL [15, 16, 17].

Personality traits have a genetic basis [18]. Recent studies indicate that genetic predispositions for neuroticism can predict psychopathology risks independent of family mental health histories [19]. Therefore, examining the links between questionnaire‐based personality traits and genetic predispositions could be an essential step in identifying factors affecting the QoL of BCS.

This research is founded on the theoretical premise that personality traits can affect psychological outcomes such as QoL, with resilience serving as a moderating variable. Additionally, genetic aspects like polygenic risk scores (PRS) for neuroticism may provide more insight into the relationship between personality traits and QoL, underscoring the necessity of considering both psychological and genetic influences.

In this study, we aimed to identify the effects of personality traits on QoL of BCS. We hypothesized that individuals' personality traits could influence the QoL of BCS. We examined five different personality traits commonly evaluated in personality research. To minimize the effects of treatment, we assessed personality traits before initiating breast cancer therapy. To elucidate the complex relationships between personality traits and QoL, we employed cluster analyses on personality dimensions. We prospectively followed participants for up to 18 months and investigated the impacts of personality measures on QoL. We also examined individual resilience to assess the influence of personality on QoL after adjusting for resilience effects. Additionally, we conducted an exploratory analysis to investigate the associations between genetic predisposition to neuroticism and the personality clusters identified in this study to ascertain whether genetic traits could be used to predict the QoL of BCS.

## 2 Methods

### 2.1 Participants

Participants were women diagnosed with primary breast cancer, scheduled for surgery at Samsung Medical Center from January 1, 2017, to August 23, 2018. Inclusion criteria included: (1) women diagnosed with primary breast cancer at stages I to III and scheduled for surgery at Samsung Medical Center, and (2) individuals who provided written informed consent for participation in this prospective cohort study. Exclusion criteria comprised: (1) patients who did not understand or agree to the written consent, (2) those diagnosed with stage IV breast cancer at the time of diagnosis, (3) individuals who did not undergo surgical treatment, (4) patients with a prior history of another cancer before the onset of breast cancer, and (5) patients who developed secondary cancer post‐breast cancer surgery.

Written informed consent was obtained from all subjects following a thorough explanation of the study. The Institutional Review Board (IRB) of Samsung Medical Center approved this study (IRB no. 2016‐07‐020).

### 2.2 Procedure

Participants completed baseline questionnaires on personality, QoL, and resilience before surgery. Follow‐up surveys evaluating QoL and resilience were administered at 6 months, 12 months, and 18 months after surgery.

### 2.3 Methods of Data Collection

Self‐report questionnaires and surveys were utilized at each visit. Basic information about the cancer (i.e., stage) and treatment methods (i.e., type of surgery and adjunctive treatment) was collected through chart reviews.

### 2.4 Measures

#### 2.4.1 Personality traits

Personality traits were assessed using the Korean version of the Big Five Inventory (BFI‐K) [20, 21]. The BFI‐K consists of 44 items distributed across five personality traits: extraversion, agreeableness, conscientiousness, neuroticism, and openness to experience. Each personality trait was measured by the sum of its corresponding positive and reversed negative items, with higher scores indicating greater levels of each trait.

#### 2.4.2 Quality of Life

The Functional Assessment of Cancer Therapy‐Breast Cancer (FACT‐B) version 4, a Korean version, was used to measure QoL [22, 23]. The FACT‐B comprises fivesubdomains: physical well‐being (PWB), social well‐being (SWB), emotional well‐being (EWB), functional well‐being (FWB), and the breast cancer subscale (BCS). Each subdomain operates independently. The PWB, SWB, and FWB each have 7 items (total scores range from 0 to 28), while EWB has 6 items (total scores range from 0 to 24). Responses were rated on a scale from 0 (‘not at all’) to 4 (‘very much’), and higher scores represent better QoL in each respective domain. The total QoL score ranges from 0 to 108, with higher scores indicating better QoL.

#### 2.4.3 Resilience

The Korean Resilience Quotient 53 (KRQ‐53) was employed to evaluate individuals' resilience [24, 25]. This instrument evaluates three major dimensions: self‐regulation, capacity for interpersonal relationships, and psychological positivity. These dimensions include nine sub‐factors: emotion regulation, impulse control, causal analysis, communication skills, empathy, ego‐resiliency, ego‐optimism, life satisfaction, and an attitude of gratitude.

#### 2.4.4 Depression and Anxiety

Depression and anxiety can also impact QoL. The Hospital Anxiety and Depression Scale (HADS) [26] was utilized to assess the severity of depression and anxiety.

### 2.5 Genotyping and PRS Analyses

In this study, genotype data were available for 355 participants. No significant differences in basic characteristics were observed between those with genotype data and those without. The Korea Biobank Array was employed for genotyping DNA samples [27]. This array, an Axiom KORV1.0‐96 Array (Affymetrix, Santa Clara, CA, USA), was designed by the Center for Genome Science at the Korea National Institute of Health. It was optimized for the Korean population and includes over 833,000 markers, which encompass common variants, rare frequency variants, and functional variants derived from sequencing data of more than 2500 Koreans. Quality control (QC) was conducted in accordance with the Korea Biobank Array protocol (http://www.koreanchip.org). The Quality control parameters for excluding study samples and variants were as follows: variants with a variant call rate < 0.99, Hardy–Weinberg equilibrium *p* < 10^−6^, minor allele frequency < 0.01 or duplicated single nucleotide polymorphisms (SNPs), samples with first‐ or second‐degree relatedness, sample call rate < 0.95, excessive heterozygosity, sex discrepancies, or outliers in principal component analysis. Genetic relatedness was inferred using KING [28]. Following sample QC and variant QC, phasing and imputation processes were carried out using Eagle v2.4 and Minimac4, utilizing the Haplotype Reference Consortium as the reference panel (Howie et al. [29]; Loh et al. [30]; McCarthy et al. [31]). Variants with imputation quality *R* ^2^ < 0.8 or a minor allele frequency < 0.01 were discarded.

Summary statistics from a genome‐wide association study for neuroticism, obtained from the Social Science Genetic Association Consortium (SSGAC) [32], were used as reference data. For neuroticism, summary statistics were compiled from a study published by the Genetics of Personality Consortium (GPC) (*N* = 63,661) combined with UK Biobank data (*N* = 107,245). The PRSs were calculated using the PRS‐CS method [33].

### 2.6 Statistical Analysis

Continuous variables are reported as mean and standard deviation, while non‐normally distributed measures are described using median and interquartile range (1st and 3rd quartiles). Categorical variables are presented as numbers and percentages.

The optimal clustering model was identified through silhouette analysis. The Wilcoxon rank sum test compared five personality subscales across clusters. Both chi‐square tests and *t*‐tests assessed differences in demographic and clinical characteristics among clusters.

Baseline QoL and resilience were analyzed across clusters, and their changes at 6 months post‐surgery were compared using the Wilcoxon rank sum test. A Generalized Estimating Equation (GEE) was employed to investigate differences in QoL changes during treatment. Univariate linear regression, adjusted for age, examined the linkage between PRS for neuroticism and neuroticism. Nagelkerke's pseudo *R* ^2^ assessed model performance, selecting the most suitable model. Additionally, PRS for neuroticism was compared across personality clusters after adjusting for age and depression. All tests were two‐sided, with a significance level set at *p* < 0.05. Except for PRS analyses, which were done using *R*, all other statistical analyses were performed with SAS version 9.4 (SAS Institute, Cary, NC, USA).

## 3 Results

Out of 506 eligible patients, 15 withdrew, leaving 491 who completed the baseline survey and underwent surgery. Eight participants were lost to follow‐up, resulting in 483 completing the six‐month post‐surgery survey. After excluding 7 patients with missing data, 476 patients were included in the analysis.

The mean age of the participants was 49.5 years (Standard Deviation [SD] = 8.8). Breast conserving surgery was performed in 64.3% of participants, and 64.7% had early‐stage disease (23.3% in Stage 0 and 41.4% in Stage I).

Average silhouette width indicated that the two‐cluster model was superior to the three‐cluster model (silhouette plot width: 0.26 for the two‐cluster model versus 0.18 for the three‐cluster model). The four‐cluster model showed even lower silhouette widths (0.20 overall and 0.16 for the poorest cluster), thus confirming the two‐cluster model as the most interpretable.

**FIGURE 1:**
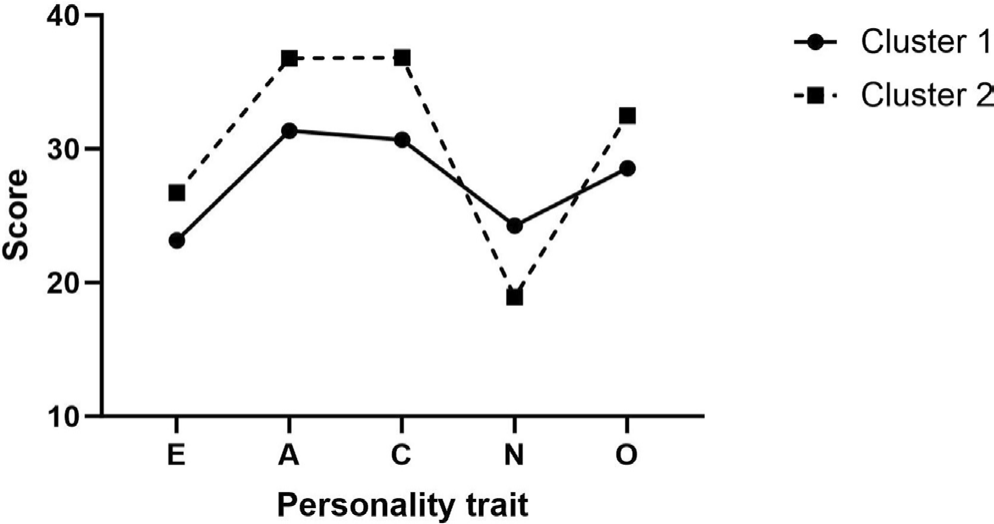
Five personality traits in two clusters. Cluster analysis was conducted using k‐means clustering. Cluster 1 (*n* = 247) exhibited higher neuroticism but lower extraversion, agreeableness, conscientiousness, and openness to experience. Conversely, Cluster 2 (*n* = 229) displayed lower neuroticism but higher extraversion, agreeableness, conscientiousness, and openness to experience.

Cluster 1 (*n* = 247) exhibited high neuroticism and lower scores on extraversion, agreeableness, conscientiousness, and openness to experience. Cluster 2 (*n* = 229) presented low neuroticism with high levels of extraversion, agreeableness, conscientiousness, and openness to experience. These patterns are illustrated in Figure 1. Significant differences were observed between the clusters across all five personality factors. Participants in Cluster 1 had a significantly higher mean age than those in Cluster 2 (50.3 years vs. 48.1 years, *p* = 0.008). Differences in surgery types were also significant, with Cluster 1 having a higher rate of total mastectomy compared to Cluster 2 (38.4% vs. 29.9%, *p* = 0.041). No significant differences were noted in other demographic and clinical variables between the clusters. Additionally, Cluster 2 exhibited higher levels of anxiety and depression symptoms than Cluster 1. Detailed results are provided in Table 1.

**TABLE 1:** Comparison of demographic and clinical features between clusters.

	All participants (*N* = 476) mean ± SD (*n*, %)	Cluster 1 (*n* = 247) mean ± SD (*n*, %)	Cluster 2 (*n* = 229) mean ± SD (*n*, %)	*p*
Age	49.2 ± 8.8	50.3 ± 8.6	48.1 ± 9.0	0.0077
Laterality				
Right	249 (52.3%)	134 (54.7%)	115 (51.3%)	0.23
Left	199 (41.8%)	97 (39.6%)	102 (45.5%)	
Both	21 (4.4%)	14 (5.7%)	7 (3.1%)	
Surgery type				
Axillary surgery alone	2 (0.4%)	—	2 (0.9%)	0.0412
Total mastectomy	161 (33.8%)	94 (38.4%)	67 (29.9%)	
Breast conserving surgery	306 (64.3%)	151 (61.6%)	155 (69.2%)	
Axillary procedure type				
No axillary surgery	32 (6.7%)	15 (6.1%)	17 (7.6%)	0.72
Sentinel lymph node biopsy	348 (73.1%)	181 (73.9%)	167 (74.6%)	
Axillary dissection	89 (18.7%)	49 (20.0%)	40 (17.9%)	
Stage of cancer				
Stage 0	111 (23.3%)	62 (25.3%)	49 (21.9%)	0.1525
Stage I	197 (41.4%)	91 (37.1%)	106 (47.3%)	
Stage II	129 (27.1%)	75 (30.6%)	54 (24.1%)	
Stage III	32 (6.7%)	17 (6.9%)	15 (6.7%)	
Chemotherapy				
Yes	219 (46.0%)	115 (47.1%)	104 (46.4%)	0.8791
No	249 (52.3%)	129 (52.2%)	120 (52.4%)	
Radiotherapy				
Yes	334 (70.2%)	167 (68.4%)	167 (74.6%)	0.1440
No	134 (28.2%)	77 (31.2%)	57 (24.9%)	
Hormone therapy				
Yes	387 (81.3%)	204 (83.6%)	183 (81.7%)	0.5853
No	81 (17.0%)	40 (16.2%)	41 (17.9%)	
Accompanied mood symptoms (mean, SD)				
Anxiety	7.1 (3.6)	6.0 (3.0)	8.2 (3.8)	< 0.001
Depression	6.0 (3.7)	4.7 (3.2)	7.4 (3.7)	< 0.001

*Note:* Cluster 1 (*n* = 247) demonstrated higher neuroticism with lower extraversion, agreeableness, conscientiousness, and openness to experience. Cluster 2 (*n* = 229) had lower neuroticism but higher extraversion, agreeableness, conscientiousness, and openness to experience.

Abbreviation: SD, standard deviation.

Differences in QoL and resilience scores at baseline and 6 months post‐surgery are detailed in Table 2. At baseline, significant differences were found between the clusters in all domains of QoL, with Cluster 1 scoring lower in all areas. Significant differences were also observed in both the total and subscale resilience scores between clusters, with Cluster 1 showing less resilience compared to Cluster 2. However, no significant differences were detected in changes to QoL or resilience 6 months post‐surgery compared to baseline between clusters. Significant associations were found between QoL variables and resilience total scores (*r* = 0.2–0.5, *p* < 0.001). No significant interaction effects were observed between QoL variables and resilience scores.

**TABLE 2:** Comparison of QoL and resilience at baseline and 6‐month post‐surgery between clusters.

Baseline	Cluster 1 (*n* = 247) mean ± SD	Cluster 2 (*n* = 229) mean ± SD	*p*
Quality of life (FACT)			
Physical well‐being	22.54 ± 4.62	24.06 ± 4.58	< 0.001
Social well‐being	17.08 ± 5.61	20.39 ± 5.59	< 0.001
Emotional well‐being	14.81 ± 4.78	17.19 ± 4.28	< 0.001
Functional well‐being	15.80 ± 5.52	20.08 ± 5.36	< 0.001
Breast cancer subscale	22.22 ± 5.1	24.76 ± 5.34	< 0.001
Functional Assessment of Cancer Therapy–Breast	92.45 ± 17.7	106.47 ± 16.36	< 0.001
Resilience (KRQ‐53)			
Total score	177.52 ± 16.42	205.36 ± 17.65	< 0.001
Self‐regulation	59.02 ± 6.54	67.59 ± 7.05	< 0.001
Interpersonal relationships	61.06 ± 6.76	70.89 ± 7.42	< 0.001
Psychological positivity	57.44 ± 7.60	66.89 ± 7.51	< 0.001

Differences at 6 months post‐surgery	Mean ± SD	Mean ± SD	*p*
Quality of life (FACT)			
Physical well‐being	−1.73 ± 4.87	−1.2 ± 6.01	0.3669
Social well‐being	−0.69 ± 5.87	−1.44 ± 6.92	0.6437
Emotional well‐being	1.64 ± 4.65	0.84 ± 4.63	0.1046
Functional well‐being	0.15 ± 5.73	−0.45 ± 6.35	0.4753
Breast cancer subscale	−0.86 ± 6.20	−1.19 ± 5.33	0.6373
Functional Assessment of Cancer Therapy‐Breast	−1.5 ± 17.26	−3.44 ± 18.66	0.3155
Resilience (KRQ‐53)			
Total score	0.66 ± 14.15	−1.67 ± 15.95	0.1021
Self‐regulation	0.08 ± 6.57	−0.43 ± 7.42	0.5015
Interpersonal relationship	0.53 ± 5.53	−0.45 ± 6.4	0.1939
Psychological positivity	0.05 ± 6.62	−0.80 ± 6.90	0.2464

*Note:* Cluster 1 (*n* = 247) demonstrated higher neuroticism but lower extraversion, agreeableness, conscientiousness, and openness to experience. Cluster 2 (*n* = 229) showed lower neuroticism but higher extraversion, agreeableness, conscientiousness, and openness to experience.

**FIGURE 2:**
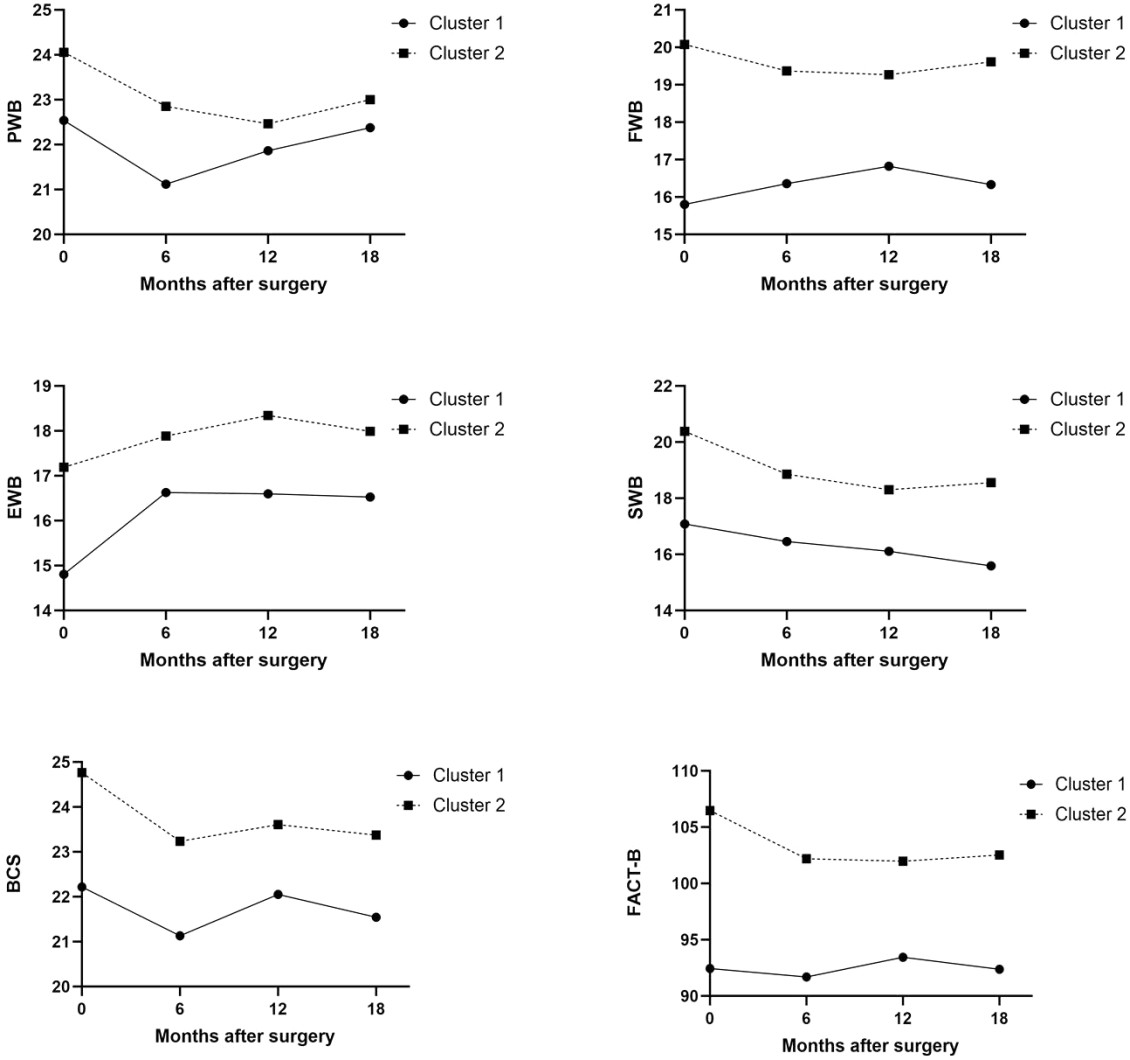
Courses of QoL changes over time between clusters. The X‐axis represents months after surgery, while the Y‐axis indicates scores of sub‐domains of the Functional Assessment of Cancer Therapy‐Breast Cancer (FACT‐B) scores. Cluster 1 (*n* = 247) exhibited higher neuroticism but lower extraversion, agreeableness, conscientiousness, and openness to experience. Conversely, Cluster 2 (*n* = 229) displayed lower neuroticism and higher levels of extraversion, agreeableness, conscientiousness, and openness to experience. BCS, Breast Cancer Subscale; EWB, emotional well‐being; FACT‐B, Functional Assessment of Cancer Therapy‐Breast; FWB, functional well‐being; PWB, physical well‐being; SWB, social well‐being.

Figure 2 illustrates the QoL at baseline and subsequent time points (6, 12, and 18 months) post‐surgery across clusters. A generalized estimating equation was used to analyze changes in QoL over time among the clusters. The differences in QoL between clusters were significant from baseline to 18 months post‐surgery. However, the interaction effect between the cluster group and the time of visit was not significant, indicating no substantial differences in QoL changes over time between clusters. The GEE analysis for FACT‐B revealed a significant main effect of Cluster (*β* = −15.0, SE = 3.83, Wald *χ* ^2^ = 15.3, *p* < 0.001), suggesting significant differences in QoL scores between Cluster 1 and 2. Although the overall effect for visit time was significant (Wald *χ* ^2^ = 27.1, *p* < 0.001), indicating that QoL changed over time, the interaction effect between cluster group and time of visit was not statistically significant (Wald *χ* ^2^ = 4.716, *p* = 0.318). Model effect tests between the cluster group and the time of visit yielded the following *p*‐values for each QoL subscale outcome: PWB (*p* = 0.104); SWB (*p* = 0.493); EWB (*p* = 0.286); FWB (*p* = 0.417); BCS (*p* = 0.348). These non‐significant findings suggest that changes in QoL during visits did not differ significantly between the two clusters.

As an exploratory analysis, PRS analyses were conducted for those with available genotype data. There were no significant differences in basic characteristics, including age and cancer‐related information, between those with and without genotype data. After age adjustment, PRS for neuroticism showed a trend towards a significant association with neuroticism (*p* = 0.066). When age and depression score were included as covariates, PRS for neuroticism was significantly associated with neuroticism (beta = 3.07, 95% confidence interval = [0.28, 5.87], *p* = 0.032). However, there were no significant differences in PRS for neuroticism between the two clusters, nor did PRSs for neuroticism significantly affect QoL measures of BCS.

## 4 Discussion

Individuals' personality traits could influence their adjustment to major life events. During the course of treatment for major illnesses, personality traits can affect commitment to care, adherence to medications, willingness to undergo certain medical decisions, reliability in follow‐up, and compliance with treatment, eventually impacting clinical outcomes [34]. Our study demonstrated that personality clusters were significantly associated with the QoL of BCS up to 18 months after diagnosis.

Some of our study participants (36 participants) underwent their final observation visit during the COVID‐19 pandemic (January and February 2020). There were no significant differences in baseline characteristics or the progression of their QoL during follow‐up visits between those who completed the observation period during the pandemic and those who did not. This may be attributed to the fact that the observation coincided with the very early stages of the pandemic (1st patient in Korea on January 20, 2020, and 1st epidemic from February to May of 2020) and Korea did not impose a lockdown during this period.

In personality clustering, a two‐cluster model proved to be more effective than a three‐cluster model. The two‐cluster model revealed that four personality traits—extraversion, agreeableness, conscientiousness, and openness to experience—exhibited similar patterns, whereas neuroticism demonstrated contrasting patterns. Prior studies have commonly endorsed three latent personality models: resilient, undercontrolled, and overcontrolled [13, 35, 36, 37]. These models typically describe high neuroticism and low extraversion as overcontrolled, analogous to cluster 2 in our study to a certain degree.

The effects of personality clusters observed in our study align well with the results of previous research [35, 36, 37]. Overcontrollers usually exhibit more internalizing problems compared to others [38]. This observation is somewhat analogous to our findings with two clusters. Previous literature on personality traits and QoL suggests that higher agreeableness, openness to experience, extraversion, and conscientiousness are typically associated with higher QoL, while increased neuroticism is inversely associated with QoL [11, 39, 40].

No significant differences were observed in QoL changes over time among clusters, nor were there significant differences in sub‐domains of QoL throughout the treatment. Previous studies have indicated that initial distress can predict psychological and physical QoL in breast cancer patients 1 year post‐baseline [7, 41, 42]. These findings suggest that early emotional distress upon diagnosis is a strong predictor of long‐term QoL.

Earlier research has shown that resilience negatively correlates with neuroticism, but positively with other personality traits: extraversion, openness to experience, agreeableness, and conscientiousness [43]. This implies that individuals with a greater tendency to form new social bonds may be protected against psychological distress [44, 45, 46]. Our study's findings also demonstrate similar resilience patterns to existing literature and indicate differences in QoL across two clusters, even when the effect of resilience is controlled.

In an exploratory analysis, we calculated PRS for neuroticism and examined its relationships within our study. Although PRS for neuroticism did not show significant associations with QoL measures, its association with study clusters suggests its potential use in predicting QoL in BCS. Further research with larger samples is required.

### 4.1 Clinical Implication

By evaluating personality and PRS for neuroticism, QoL of BCS can be anticipated even before treatment commences. Behavioral interventions targeting neuroticism have proven effective in reducing negative affect and enhancing positive affect [47]. Similarly, a tailored approach including psychosocial interventions could be adopted for populations predicted to experience lower QoL based on their initial assessments prior to treatment. Moreover, genetic studies could yield further evidence helpful in determining additional interventions to enhance QoL. Future research incorporating targeted interventions for vulnerable groups is justified.

### 4.2 Limitations

Our study has several limitations. Firstly, its small sample size may influence the findings. Additionally, we lacked independent validation samples, which could compromise the accuracy of PRS analysis. Secondly, as all participants were recruited from a single tertiary care hospital, there may be a potential bias in participant selection. Previous studies have shown that patients with metastatic breast cancer generally exhibit poorer QoL than those without metastasis [48]. Accordingly, we excluded patients with metastatic breast cancer, limiting the generalizability of our findings to all breast cancer patients. Thirdly, the 18‐month follow‐up period may be insufficient to fully assess the effects of personality traits on QoL. A longer‐term study is required. Fourthly, we utilized GWAS summary data from individuals of European ancestry as reference data. Using summary data from a different ancestry could reduce effect sizes. Nonetheless, no large‐scale GWAS data for neuroticism in the East Asian population were publicly available. Fifthly, our results do not establish a causal relationship between personality dimensions and QoL.

## 5 Conclusions

Our research indicates that personality clusters significantly influence the long‐term QoL of BCS, with differences persisting up to 18 months post‐diagnosis. Moreover, neuroticism measured in our study is associated with genetic predispositions for neuroticism assessed through PRS. Future investigations including personalized interventions to improve the QoL of BCS based on their personality clusters are warranted.

## Author Contributions

**In Mok Song:** formal analysis (equal), writing – original draft (lead), writing – review and editing (equal). **Eun Young Cho:** project administration (equal), resources (equal). **Ji Hyun Baek:** conceptualization (equal), funding acquisition (equal), methodology (equal), writing – review and editing (lead). **Se Kyung Lee:** conceptualization (equal), investigation (equal), methodology (equal), supervision (equal), writing – review and editing (equal).

## Ethics Statement

All procedures contributing to this work adhered to the ethical standards of the relevant national and institutional committees on human experimentation and complied with the Helsinki Declaration of 1975, as revised in 2008.

## Conflicts of Interest

The authors declare no conflicts of interest.

## Data Availability

The data supporting the findings of this study are available from the corresponding author upon reasonable request.
